# Artificial intelligence for the detection and diagnosis of oral and maxillofacial lesions: evidence, limitations, and future directions

**DOI:** 10.1590/1807-3107bor-2026.vol40suppl1058

**Published:** 2026-08-24

**Authors:** Maria Augusta VISCONTI, Michael Marc BORNSTEIN, Alan Roger SANTOS-SILVA, Manoela Domingues MARTINS, Matheus Lima OLIVEIRA

**Affiliations:** (a) Universidade Federal do Rio de Janeiro – UFRJ, School of Dentistry, Department of Pathology and Oral Diagnosis, Rio de Janeiro, RJ, Brazil; (b) University of Basel, University Center for Dental Medicine Basel UZB, Department of Oral Health & Medicine, Basel, Switzerland; (c) Universidade Estadual de Campinas – Unicamp, Piracicaba Dental School, Department of Oral Diagnosis, Piracicaba, SP, Brazil; (d) Universidade Federal do Rio Grande do Sul – UFRGS, School of Dentistry, Department of Oral Pathology, Porto Alegre, RS, Brazil

**Keywords:** Artificial Intelligence, Odontogenic Cysts, Odontogenic Tumors, Precancerous Conditions, Diagnosis, Computer-Assisted

## Abstract

Artificial intelligence (AI) has emerged as a promising tool to support or perform specific oral diagnostic tasks, particularly through advances in machine learning and deep learning. This comprehensive review aimed to synthesize current evidence and explore future directions for AI-driven or AI-supported oral diagnostic workflows across four target pathologies: odontogenic cysts, odontogenic tumors, oral potentially malignant disorders (OPMDs), and oral squamous cell carcinoma (OSCC). A structured search strategy combining MeSH terms and free-text keywords was applied, encompassing target conditions, AI methodologies, diagnostic performance metrics, and clinician comparator groups. Across all pathologies, AI models demonstrated encouraging performance, frequently approaching that of experienced clinicians, particularly in image-based detection and classification tasks. In some contexts, improved sensitivity was observed, suggesting potential value in early disease detection. However, findings were highly variable and often limited by methodological constraints, including retrospective study designs, small or curated datasets, lack of external validation, and heterogeneity in reporting metrics such as sensitivity, specificity, accuracy, and area under the curve. Importantly, no consistent evidence supports the superiority of AI over clinicians across all performance measures. Instead, current data suggest that AI may serve as a valuable adjunct to clinical decision-making, with potential to reduce diagnostic variability and support non-specialist practitioners. Future research should prioritize prospective, multicenter studies with standardized methodologies, robust external validation, and evaluation of real-world and patient-centered outcomes. While AI holds significant promise, its routine clinical implementation for oral diagnostic tasks remains premature, requiring further validation, transparency, and integration into clinical workflows.

## Introduction

Artificial intelligence (AI) has rapidly emerged as a transformative technology in healthcare, including assisting in the detection and diagnosis of various oral diseases. Broadly defined as the ability of computational systems to perform tasks that typically require human intelligence, AI encompasses sub fields such as machine learning (ML) and deep learning (DL). ML refers to algorithms that learn patterns from data to generate predictions, whereas DL, a subset of ML, employs multilayered neural networks capable of processing complex imaging and clinical datasets.^1^, ^2^

In oral medicine, oral and maxillofacial pathology, and oral and maxillofacial radiology, AI has shown increasing potential across multiple domains. This comprehensive review synthesizes current evidence on the performance of AI in assisting the diagnosis of four target pathologies: odontogenic cysts, odontogenic tumors, oral potentially malignant disorders (OPMDs), and oral squamous cell carcinoma (OSCC). Despite notable advances, the current body of evidence remains fragmented across different disease types, methodologies, and performance metrics. Therefore, a comprehensive and critically structured synthesis of the available literature is warranted to consolidate existing knowledge, identify gaps, and provide a clearer framework for future research.

To ensure methodological rigor and transparency, this review was structured according to the Scale for the Assessment ofNarrative Review Articles (SANRA), a validated tool for the critical appraisal of non-systematic reviews.^3^ The manuscript is organized into thematic sections with integrated critical discussion, allowing for a continuous synthesis and appraisal of the evidence within each target pathology. By integrating current evidence and methodological considerations, the aim of this review was to provide a comprehensive overview of the present scientific landscape and highlight future directions for AI-driven oral diagnostic workflows.

### Search strategy

A structured literature search was conducted in MEDLINE (via PubMed) to identify studies evaluating the comparative diagnostic performance of AI-based tools and clinicians in the detection of odontogenic cysts, odontogenic tumors, OPMDs, and OSCC. The search strategy combined controlled vocabulary (MeSH terms) and free-text keywords related to four core domains: a) the target condition (odontogenic cysts, odontogenic tumors, OPMDs or OSCC), b) AI and ML methods, c) diagnostic performance metrics, and d) clinician or human comparator groups (Table).

**Table:** Search strategy components: domains, MeSH terms, and keywords used for this critical review.

Search domain	Controlled vocabulary (MeSH Terms)	Free-text keywords	Boolean operator
#1 Target Condition (Pathology)	“Odontogenic Cysts”[MeSH]	odontogenic cyst, periapical cyst, radicular cyst, dentigerous cyst, odontogenic keratocyst, Adenomatoid Odontogenic Tumor, Calcifying Epithelial Odontogenic Tumor, Pindborg Tumor, Odontogenic Myxoma, Oral Potentially Malignant Disorders, erythroplakia, oral epithelial dysplasia, oral premalignant lesions, Oral Squamous Cell Carcinoma, oral cancer	**OR** (within domain)
	“Odontogenic Tumors”[MeSH]		
	“Ameloblastoma”[MeSH]		
	“Mouth Neoplasms”[MeSH]		
	“Carcinoma, Squamous Cell”[MeSH]		
	“Leukoplakia, Oral”[MeSH]		
#2 AI/ML Methods	“Artificial Intelligence”[MeSH]	convolutional neural network, AI, ML, DL, CNN	**OR** (within domain)
	“Machine Learning”[MeSH]		
	“Deep Learning”		
	“Neural Networks, Computer”[MeSH]		
	“Image Interpretation, Computer-Assisted”[MeSH]		
#3 Diagnostic Performance Metrics	“Sensitivity and Specificity”[MeSH]	ROC, AUC	**OR** (within domain)
	“Diagnostic Accuracy”[MeSH]		
	“Receiver Operating Characteristic Curve”[MeSH]		
	“Early Detection of Cancer”[MeSH]		
#4 Comparator Group (Human)	“Dentists”[MeSH]	clinicians, radiologists, experts, human performance	**OR** (within domain)
	“Physicians”[MeSH]		
	“Pathologists”[MeSH]		
	“Radiologists”[MeSH]		
#5 Ethics and Legal Aspects*	“Ethics”[Mesh]	transparency, accountability, legal liability, algorithmic bias, data privacy	**OR** (within domain)
	“Ethics, Dental”[Mesh]		
	“Social Responsibility”[Mesh]		
Final Strategy		**#1 AND #2 AND #3 AND #4** (supplemented by **#5** for qualitative analysis)	

Condition-related terms were defined for each target pathology, including “Odontogenic Cysts” [MeSH], odontogenic cyst, periapical cyst, radicular cyst, dentigerous cyst, and odontogenic keratocyst for odontogenic cysts and “Odontogenic Tumors” [MeSH], “Ameloblastoma” [MeSH], Adenomatoid Odontogenic Tumor, Calcifying Epithelial Odontogenic Tumor, Pindborg Tumor, Odontogenic Myxoma for odontogenic tumors. For OPMDs, the condition-related terms included “Oral Potentially Malignant Disorders”, “Oral Precancerous Conditions”[MeSH], “Precancerous Conditions”[MeSH], “Leukoplakia, Oral”[MeSH], erythroplakia, oral epithelial dysplasia, and oral premalignant lesions. For OSCC, the search incorporated “Oral Squamous Cell Carcinoma”, “Mouth Neoplasms”[MeSH], “Carcinoma, Squamous Cell”[MeSH], and oral cancer. Within each target pathology, all terms were combined using the Boolean operator OR.

AI-related terms included “Artificial Intelligence”[MeSH], “Machine Learning”[MeSH], “Deep Learning”, “Neural Networks, Computer”[MeSH], “Image Interpretation, Computer-Assisted”[MeSH], convolutional neural network, AI, ML, DL, and CNN. Diagnostic performance concepts were captured using “Sensitivity and Specificity” [MeSH], “Diagnostic Accuracy”, “Receiver Operating Characteristic” [MeSH], ROC, AUC, and “Early Detection ofCancer” [MeSH]. Comparator terms included clinicians, dentists [MeSH], physicians [MeSH], pathologists [MeSH], radiologists, experts, and human performance. To address the ethical and legal dimensions of these technologies, the domains were further intersected with terms such as “Ethics” [Mesh], “Ethics, Dental” [Mesh], “Social Responsibility” [Mesh], transparency, accountability, legal liability, algorithmic bias, and data privacy.

The four core domains were combined using the Boolean operator AND to retrieve studies directly comparing AI-based diagnostic systems with clinician performance, in cases of odontogenic cysts, odontogenic tumors, suspected OPMDs or OSCC, while the supplementary ethical search supported the qualitative analysis of the legal implications in clinical practice. No initial restrictions were placed on study design to capture comparative test accuracy studies, diagnostic cohort studies, and validation studies. Reference lists of relevant systematic reviews were screened manually to identify additional eligible studies.

## Critical discussion of the literature

The conceptual framework of AI for the detection and diagnosis of the four target pathologies (i.e., odontogenic cysts, odontogenic tumors, OPMDs, and OSCC) is summarized in Figure, which depicts the clinical challenges, input data, main AI approaches, tasks, performance metrics, comparative performance relative to clinicians, clinical role, limitations, and future directions.

### AI for detection and diagnosis of odontogenic cysts

The detection, diagnosis, and, consequently, management of odontogenic cysts may undergo significant transformations with the integration of AI. These lesions, which primarily include periapical (radicular) cysts, dentigerous cysts, and odontogenic keratocysts (OKCs), present diagnostic challenges due to their varied radiographic and histopathological features. Importantly, these entities differ not only in their biological behavior but also in their clinical and surgical implications. While inflammatory lesions such as periapical cysts are generally less aggressive and often managed conservatively, OKCs are associated with a more aggressive growth pattern, a higher recurrence rate, and greater potential for bone destruction. In this context, diagnostic inaccuracies, particularly false-negative results for more aggressive cystic entities, may lead to inadequate treatment planning and adverse clinical outcomes, highlighting the need for reliable and interpretable AI-assisted decision-making.

Deep learning, particularly convolutional neural networks (CNNs), has been applied to panoramic radiography and cone-beam computed tomography (CBCT) for cyst detection and diagnosis. Importantly, detection refers to identifying the presence and location of a lesion, whereas classification (i.e., diagnosis) involves correctly assigning the lesion to a specific pathological category. A modified nnU-Net CNN model has been developed for classifying and segmenting five types of jaw lesions on CBCT, where segmentation refers to the precise delineation of lesion boundaries from surrounding tissues, achieving F1-scores of 0.923 for periapical cysts and 0.857 for dentigerous cysts.^4^ The F1 score provides an overall measure of how well a model correctly identifies cases with a pathology while avoiding incorrect classifications by integrating both missed cases and false positives into a single value. Another CNN algorithm has also been shown to achieve high accuracy for both dentigerous and periapical cysts on CBCT scans.^5^ Similarly, the YOLO v11 CNN architecture applied to panoramic radiographs has demonstrated an average precision of 95% for the detection of dentigerous cysts and 85% for OKCs.^6^ Collectively, these findings underscore the potential of DL to assist clinicians in detecting, classifying, and segmenting cystic lesions.

The application of AI has also extended to the evaluation of histopathological images to assist in the definitive diagnosis. Several DL models have been evaluated to assess histopathological features of odontogenic cysts, with VGG16 and VGG19 CNN models achieving Fl-scores of 0.89 and outperforming other architectures.^7^ This approach may reduce interobserver variability typically seen even between trained pathologists. For the differential diagnosis of specific jaw pathologies, radiomics and inflammatory biomarkers have been combined in an ML-based model to distinguish OKCs from dentigerous cysts, achieving 94.3% accuracy.^8^

**Figure:**
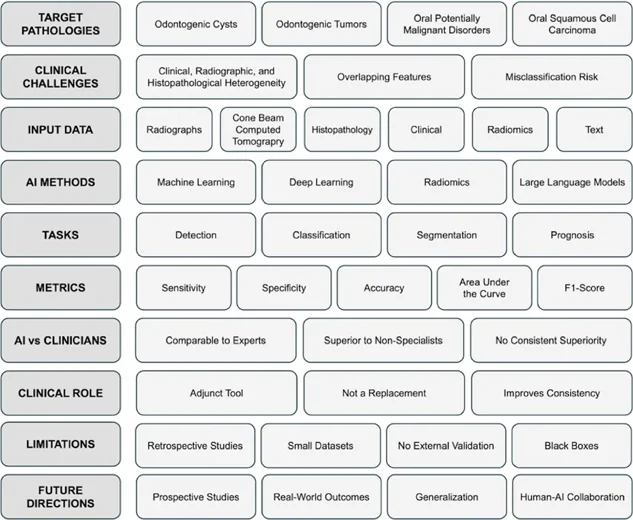
Conceptual framework of artificial intelligence (AI) applications in the detection and diagnosis of odontogenic cysts, odontogenic tumors, oral potentially malignant disorders, and oral squamous cell carcinoma.

Recent innovations include transformer-based models and hybrid architectures. Chen et al.^9^ applied Swin-UNETR for periapical lesion segmentation on CBCT, demonstrating the utility of pretrained transformers with limited datasets. Mohanty et al.^10^ developed a hybrid encoder iterative attention convolution model to predict OKC recurrence from whole-slide histopathology images, achieving a perfect area under the receiver operating characteristic curve (AUC) of 1.0 and 96% recall, meaning that nearly all actual recurrent cases were correctly identified based on the initial histological specimen analysis. These advanced models illustrate Al’s expanding role beyond mere detection or diagnosis of pathology, extending into prognostic applications such as recurrence prediction.

The use oflarge language models (LLMs) such as ChatGPT has been explored for radiographic interpretation. Variable and clinically limited diagnostic performance for jaw lesions on panoramic radiographs has been reported.^11^, ^12^ However, a systematic review found that multimodal LLMs can serve as adjunctive tools, with diagnostic accuracy rates ranging from 25% to 96% depending on factors such as the model version, the type of input modality (e.g., text and visual data, including clinical descriptions, photographic images, and histopathology reports), and the complexity of the lesion being assessed.^13^ A concordance rate of 41.4% with histopathology for OKCs has also been reported for ChatGPT-4, which is slightly lower than the rates obtained by clinicians and a Bayesian diagnostic tool, which uses probabilistic reasoning based on prior data and clinical findings.^14^

A systematic review including periapical radiographs, panoramic radiographs, CBCT, and multidetector computed tomography concluded that evidence for the routine clinical application of ML in jaw lesions remains inconclusive, with methodological flaws such as lack of external validation cited as major limitations.^15^ Similarly, another systematic review found that DL models for periapical lesion detection in panoramic radiographs have shown moderate to high performance but are not yet considered ready for definitive clinical practice.^16^ Many studies lack histopathological correlation, and heterogeneity in datasets and evaluation metrics limits comparability.

Given the current body of evidence, AI is not yet ready for routine clinical application in the detection, diagnosis and management of odontogenic cysts. While promising results have been demonstrated across various imaging modalities and histopathological analyses, performance remains variable, and methodological limitations preclude definitive conclusions regarding its applicability in routine daily practice.

### AI for detection and diagnosis of odontogenic tumors

The diagnostic transition from odontogenic cysts to tumors is marked by increased complexity, firstly because these lesions are less common than jaw cysts, and secondly because epithelial and mesenchymal odontogenic neoplasms may present with radiographic features like those of cystic lesions, such as OKCs. While early AI applications predominantly focused on detection and segmentation tasks, such approaches are insufficient for odontogenic tumors, where diagnostic performance must extend beyond simple localization to reliable differential classification. Indeed, lesions such as ameloblastoma, adenomatoid odontogenic tumor (AOT), and odontogenic myxoma demand superior specificity due to their invasive biological behavior and distinct therapeutic requirements. This distinction is clinically consequential, as these tumors often necessitate extensive surgical management, underscoring the imperative for AI models that function as robust decision-support tools rather than mere identification systems.^17^, ^18^

The integration of AI in this field is currently focused primarily on automating image-based detection and diagnosis using panoramic radiography and CBCT. Among odontogenic tumors, ameloblastoma has been the most extensively investigated, largely due to its clinical prevalence and its potential for local invasion and recurrence. Recent systematic reviews underscore this trend, demonstrating the high performance of DL models in detecting ameloblastomas on panoramic radiographs. Current evidence suggests that YOLO-based CNN models achieve an accuracy of approximately 89.3% for classifying and differentiating ameloblastomas and OKCs on panoramic radiographs. In addition to detection, there is a growing emphasis on automated segmentation, enabling more detailed volumetric assessment of these tumors.^18^

Recent evidence confirms that DL architectures, such as Mask R-CNN, effectively delineate odontogenic tumors, specifically ameloblastomas, on multidetector CT and CBCT scans. Notably, these models maintain high diagnostic efficacy even when trained on limited datasets, demonstrating robust performance suitable for resource-constrained clinical settings.^19^ This capability significantly advances surgical planning by providing reliable volumetric measurements of tumor expansion and cortical bone involvement. Such precise segmentation is critical for determining optimal surgical margins and forecasting potential complications, thereby facilitating accurate, individualized clinical decision-making.^19^, ^20^

Current literature reveals that diagnostic accuracy remains inconsistent across odontogenic tumor types. While ameloblastoma yields robust results due to its higher prevalence, rarer entities such as AOT and odontogenic myxomas continue to pose significant challenges.^17^ The scarcity of training data for these infrequent lesions often reduces predictive performance, underscoring that the generalizability to clinical practice remains contingent upon the development of large-scale, multicenter datasets.^18^

Despite data heterogeneity, radiomics significantly reduces the subjectivity of conventional visual interpretation. By extracting quantitative features like a pixel distribution and intralesional heterogeneity, imperceptible to the human eye, this technology identifies diagnostic signatures, including the “tennis racket” pattern of odontogenic myxoma and internal calcifications in calcifying epithelial odontogenic tumors. Machine learning models using XGBoost and CBCT-based radiomics have demonstrated robust performance in differentiating ameloblastoma from OKCs, achieving an F1-score of 0.841.^20^ Furthermore, this ‘virtual biopsy’ paradigm enables non-invasive molecular profiling, such as predicting BRAF-V600E mutations in ameloblastomas, directly supporting risk stratification and targeted therapy.^21^

Beyond radiography, whole slide imaging represents an expanding frontier in odontogenic tumor diagnostics. CNNs can now classify histological variants with accuracy comparable to that of experienced pathologists, significantly reducing interobserver variability. Transfer learning, utilizing architecture pretrained on large-scale datasets like ImageNet, optimizes diagnostic reliability even when applied to limited multicenter data.^15^ This approach is essential for distinguishing entities with overlapping histological patterns, such as AOT, ameloblastomas, and ameloblastic carcinomas. Furthermore, AI enables the identification of specific cellular features linked to aggressive behavior, transitioning digital pathology from basic classification toward predictive, personalized clinical management.^15^, ^17^

Despite recent progress, the clinical implementation of AI tools in odontogenic tumor detection, diagnosis, and management remains hindered by significant methodological hurdles. Current evidence primarily stems from retrospective studies utilizing curated datasets, which may artificially inflate model performance and introduce selection biases absent in real-world diagnostic settings.^18^, ^19^ Consequently, there is a distinct lack of robust external validation; algorithms achieving high internal performance often exhibit poor generalizability when applied to diverse patient populations or varying imaging protocols.^15^, ^20^ Finally, the ‘black-box’ nature of most DL models, characterized by their inability to provide clear, human-interpretable explanations for their outputs, limits the transparency of algorithmic decisions. This opacity sustains ethical concerns and hinders the clinical trust of end users (e.g. clinicians in daily practice) required for definitive adoption.^17^, ^22^

### AI for detection and diagnosis of OPMDs

The clinical evaluation of OPMDs remains challenging due to their heterogeneous clinical presentation and the broad spectrum ofunderlying epithelial alterations, which may range from benign inflammatory conditions and disturbances of epithelial maturation to varying degrees of dysplasia or even established malignant transformation. In this scenario, AI may serve as a supportive tool to assist clinicians in the assessment of suspected OPMDs. The present section focuses specifically on the comparative diagnostic performance of ML-based tools and experienced clinicians when evaluating suspected OPMDs. The central question is not whether AI systems are accurate in absolute terms, but whether they surpass, equal, or fall short of expert human judgment under comparable testing conditions.

Current evidence indicates that ML systems can achieve diagnostic performance metrics that are at least comparable to those of experienced specialists and consistently superior to those of non-specialists. In a two-stage DL model for clinical OPMD classification, Cao et al.^23^ demonstrated F1 scores ranging from 89.9% to 92.2%, significantly outperforming junior, intermediate, and senior clinicians whose F1 scores ranged from 77.7% to 82.6% on the same image sets. Notably, when clinicians were assisted by AI outputs, junior examiners achieved performance levels comparable to senior clinicians, highlighting the augmentation potential of ML tools rather than a purely competitive replacement paradigm. These findings suggest that ML may elevate generalist performance toward specialist standards, which is of relevance in settings with limited access to oral medicine expertise.

Similarly, Schwárzler et al.^24^ evaluated a YOLOv8-based model trained across eleven oral mucosal categories, including benign lesions, OPMDs, and oral cancer. On shared test images, the ML system performed comparably to oral surgery specialists (p = 0.93) and significantly better than general dentists (p < 0.01). This head-to-head comparison highlights an important nuance: ML systems demonstrate non-inferiority to experts and superiority to general practitioners, but do not consistently demonstrate statistically significant superiority over specialists. These data align with earlier findings in broader neural network studies, in which diagnostic accuracy reached 92.3% for ML models versus 92.4% for expert panels, again demonstrating parity rather than dominance.^25^

Systematic reviews and meta-analyses further contextualize these comparative findings. Pooled sensitivities for AI-based imaging in OPMDs and oral cancer range approximately from 0.87 to 0.92, reflecting the proportion of true positive cases correctly identified, with specificities between 0.81 and 0.89, reflecting the proportion of true negative cases correctly ruled out, and AUC values frequently exceeding 0.94.^25^, ^26^, ^27^, ^28^ These estimates are within the range reported for specialist visual examination, suggesting equivalence in controlled settings. However, as emphasized in comparative accuracy reviews, including the work of Nieri et al.,^29^ direct head-to-head prospective trials remain limited and frequently exhibit methodological concerns such as retrospective image selection, spectrum bias, and lack of external validation. Moreover, predictive modelling for malignant transformation risk in OPMDs remains an evolving field, with ML approaches showing promise but not yet demonstrating consistent real-world superiority over experienced clinical risk stratification.^30^

Taken together, the available evidence suggests that ML-based diagnostic tools for suspected OPMDs are at least non-inferior to experienced specialists in image-based diagnostic tasks and clearly superior to non-specialists. Nonetheless, robust proof of consistent superiority over expert clinicians in real-world, prospective clinical practice is currently lacking. The most evidence-supported use case is therefore integration as assistive tools capable of reducing diagnostic variability, improving generalist performance, and enhancing early referral pathways, rather than the replacement of specialist clinical judgment. Future comparative studies should prioritize prospective, multicenter designs with standardized reference standards and clinician-AI interaction frameworks to more definitively determine whether ML can exceed expert-level diagnostic performance in routine care.

### AI for detection and diagnosis of oral cancer

The comparative diagnostic performance of ML-based tools and experienced clinicians in patients with suspected OSCC, the most common type of oral cancer, represents a critical and evolving area of research in oncology. The present manuscript specifically addresses whether ML systems achieve superior diagnostic accuracy compared with expert human examiners when evaluating primary OSCC. This question extends beyond establishing high standalone performance of AI models and instead interrogates their relative performance against clinical expertise under comparable diagnostic conditions.

Meta-analyses across multiple diagnostic modalities consistently report high pooled sensitivity and specificity for AI-based systems applied to OSCC detection. Across photographic images, histopathology, and radiologic imaging, pooled sensitivity and specificity estimates generally range between approximately 0.92 and 0.95, suggesting robust discriminatory capacity.^31^, ^32^, ^33^, ^34^ These values demonstrate that ML models can accurately identify malignant lesions when benchmarked against histopathological confirmation. However, most of these analyses compare AI outputs to biopsy-proven diagnoses rather than directly to clinician performance, limiting inference regarding superiority over human experts. In other words, while AI demonstrates excellent accuracy relative to gold-standard pathology, the crucial question of comparative performance versus experienced clinicians requires more focused examination.

Direct comparative evidence suggests that ML systems are generally non-inferior to experienced clinicians but not consistently superior. A comparative test-accuracy systematic review evaluating DL systems for clinical oral cancer detection found no clear advantage of AI over human experts. The pooled sensitivity difference between AI and clinicians was 0.024, indicating that AI had approximately 2.4% higher sensitivity (95%CI: −0.093 to 0.206), while the specificity difference indicated that AI had approximately 4.1% lower specificity than clinicians (95%CI: −0.218 to 0.038), indicating statistical equivalence within wide confidence intervals and low-certainty evidence.^29^ These findings underscore that, although AI may demonstrate slightly higher sensitivity in certain datasets, specificity frequently remains similar or marginally favors clinicians, and overall superiority is not consistently demonstrated.

Similar findings are reported by Fu et al.,^35^ who demonstrated that a DL algorithm applied to photographic images of the oral cavity achieved high sensitivity for OSCC detection, comparable to that of clinicians, with potential improvements in speed and standardization. Similarly, histopathology-based DL models have shown diagnostic performance approaching that of experienced pathologists.^36^ In experimental settings, DL classifiers have demonstrated high concordance with pathologist interpretation in histopathological OSCC diagnosis.^37^ However, these studies typically emphasize equivalence or slight improvements in sensitivity rather than consistent, statistically significant superiority across both sensitivity and specificity metrics.

An important distinction emerges when considering staging rather than primary lesion detection. In the context of predicting lymph node metastasis using CT and MRI, AI models have demonstrated AUC values around 0.92 and, in some analyses, outperformed radiologists overall.^38^ Nevertheless, this pertains to nodal staging rather than initial detection of primary OSCC and thus does not directly answer the question of diagnostic superiority for primary tumor identification.

From a clinical perspective, the translational significance of these findings is nuanced. Across studies, AI systems appear at least non-inferior to experienced clinicians and occasionally demonstrate modest gains in sensitivity. However, specificity may be comparable or slightly lower, and the overall evidence base remains constrained by methodological limitations. Common concerns include small sample sizes, retrospective study designs, spectrum bias due to enriched case datasets, heterogeneity in imaging modalities, and reliance on internal validation rather than independent external cohorts.^29^, ^31^, ^34^, ^35^ These limitations reduce certainty regarding real-world generalizability and prevent definitive claims of superiority over expert clinical judgment.

The strongest and most encouraging finding emerging from the literature is that ML tools function most effectively as adjunctive technologies. AI assistance has the potential to enhance clinician sensitivity, reduce inter-observer variability, standardize diagnostic thresholds, and accelerate image interpretation, particularly in high-volume or resource-limited settings.^35^, ^36^, ^37^ Rather than replacing experienced clinicians, ML systems appear to support a model of collaborative intelligence in which algorithmic pattern recognition complements human contextual reasoning, including integration of patient history, tactile findings, and longitudinal lesion evolution- factors not always captured in static imaging datasets.

In summary, current evidence indicates that ML-based diagnostic tools for suspected OSCC achieve performance comparable to that of experienced clinicians and, in certain contexts, may demonstrate slightly improved sensitivity. However, consistent and robust superiority across sensitivity, specificity, and overall accuracy has not been conclusively established. The available data support the interpretation of AI as a high-performing adjunctive tool pending rigorous prospective, multicenter, externally validated comparative trials. Future research should prioritize real-world implementation studies evaluating patient-centered outcomes such as time to diagnosis (reduction of professional delay), stage at detection, and reduction in missed cancers to more definitively determine whether ML can surpass expert human diagnostic performance in routine clinical practice.

## Ethical implications of AI implementation

The integration of AI into clinical dentistry transcends mere technical optimization and necessitates a critical re-examination of its bioethical foundations.^39^ While AI offers significant potential to enhance diagnostic accuracy, its implementation must be carefully balanced against systemic risks such as algorithmic bias and the inherent opacity of black-box models, which often stem from narrow training datasets and a lack of robust external validation.^40^ Within this framework of augmented intelligence, patient autonomy requires that individuals be clearly informed about the role and limitations of AI. This ensures that the clinician’s interpretive expertise remains the final safeguard against diagnostic error and that accountability remains unequivocally human.^40^

Diagnostic models trained predominantly on homogeneous datasets from the Global North may fail to capture the biological diversity of admixed populations, such as those in Brazil, risking the reinforcement of racial and ethnic disparities under the guise of mathematical neutrality.^41^ Addressing this issue requires more than expanding datasets; it necessitates strict adherence to reporting standards such as STARD-AI and TRIPOD-AI. These frameworks promote transparency and reproducibility while fostering the inclusion of underrepresented groups, thereby mitigating the risk of ‘algorithmic ghosting’.^42^

The growing reliance on large-scale datasets necessitates the exchange of substantial volumes of radiographic and clinical images, making strict compliance with regulatory frameworks, such as the General Data Protection Regulation (GDPR) and the Brazilian General Data Protection Law (LGPD), essential. Beyond conventional de-identification, safeguarding patient metadata has become a critical ethical responsibility to mitigate the risk of sensitive data exposure.^41^, ^43^ In this context, data security transcends mere regulatory compliance, establishing itself as a fundamental pillar of clinical trust and holistic patient protection.^41^

Finally, the emergence ofGenerative AI (GenAI) introduces a dual challenge. While these tools enhance efficiency in administrative and clinical workflows,^44^ their susceptibility to factual hallucinations poses a direct risk to patient safety.^45^, ^46^ Consequently, the ‘human-in-the-loop’ principle must be understood not only as a technical safeguard but as a non-transferable professional responsibility. Clinical judgment should not merely prevail over algorithmic outputs; it must actively scrutinize them, ensuring that the clinician remains the ultimate moral and legal authority in the diagnostic process.^46^

## Conclusion

Artificial intelligence has demonstrated substantial potential across multiple domains of detection and diagnosis ofvarious oral diseases, including odontogenic cysts, odontogenic tumors, oral potentially malignant disorders, and oral squamous cell carcinoma. Across these conditions, AI systems, particularly those based on deep learning, have achieved performance that approaches that of experienced clinicians, with occasional improvements in specific metrics such as sensitivity. However, these findings must be interpreted with caution. The current body of evidence is characterized by important methodological limitations, including reliance on retrospective and highly curated datasets, heterogeneity in study design, inconsistent reporting of diagnostic performance metrics, and a lack of robust external validation.

In summary, the available data do not yet support the routine clinical adoption of AI as an independent detection and diagnostic tool for various oral pathologies. Rather, AI should currently be regarded as a promising adjunct capable of supporting clinical decision-making, reducing diagnostic variability, and potentially enhancing early detection pathways, particularly among non-specialist practitioners.

## Data Availability

The datasets generated during and/or analyzed during the current study are available from the corresponding author on reasonable request.
